# Supplementary material for: Two-dimensional electrons at mirror and twistronic twin boundaries in van der Waals ferroelectrics
Source: Nat Commun. 2024 Aug 9;15:6838. doi: 10.1038/s41467-024-51176-1 (PMC11316064; doi:10.1038/s41467-024-51176-1)
Supplement: Supplementary file 1 — Supplementary Information [file 41467_2024_51176_MOESM1_ESM.pdf]

Supplementary Information:
Two-dimensional electrons at mirror and twistronic twin boundaries in van der Waals ferroelectrics

James G. McHugh,[1,2] Xue Li,[1,2] Isaac Soltero,[1,2] and Vladimir I. Fal'ko[1,2,3,*]

[1] *Department of Physics and Astronomy, University of Manchester. Oxford Road, Manchester, M13 9PL, United Kingdom*
[2] *National Graphene Institute, University of Manchester. Booth St. E., Manchester, M13 9PL, United Kingdom*
[3] *Henry Royce Institute for Advanced Materials, University of Manchester, Oxford Road, Manchester, M13 9PL, United Kingdom*

Supplementary Table I. **DFT-calculated bulk TMD parameters** (Left) DFT-optimised and experimental structural parameters (in-plane: a, and out of plane: c lattice constants) of 2H and 3R bulk TMDs. (Right) Intrinsic electric field and effective masses of electrons and holes. m_z is calculated from direct fitting of band dispersion in periodic 2H and by fitting dispersion of carriers in a triangular quantum well to mTB band structure.

		a [Å]	c [Å]	a (expt) [Å]	c (expt) [Å]		$\lvert \mathbf{E}_{\mathrm{FE}} \rvert$ [V/nm]	$m_x/m_y/m_z$ [m_0]	Δ_{SO} [meV]
MoS_2	2H	3.151	12.242	3.160 [1]	12.294 [1]	e_Q	0.115	0.51/0.75/0.51	31
	3R	3.154	12.158	-	-	h_Γ		-0.67/-0.67/-0.88	0
WS_2	2H	3.155	12.306	3.153 [2]	12.324 [2]	e_Q	0.110	0.53/0.56/0.48	150
	3R	3.158	12.266	3.158 [2]	12.272 [2]	h_Γ		-0.61/-0.61/-0.75	0
$MoSe_2$	2H	3.281	12.936	3.289 [1]	12.928 [1]	e_Q	0.092	0.48/0.71/0.50	12
	3R	3.286	12.890	-	-	h_Γ		-0.83/-0.83/-1.30	0
WSe_2	2H	3.282	12.982	3.282 [2]	12.962 [2]	e_Q	0.091	0.45/0.52/0.43	67
	3R	3.287	12.960	-	-	h_Γ		-0.74/-0.74/-1.10	0
$MoTe_2$	2H	3.580	14.244	3.519 [3]	13.964[3]	e_Q	0.079	0.484/1.33/0.55	22
	3R	3.589	14.256	-	-	h_Γ		-1.14/-1.14/-2.09	0

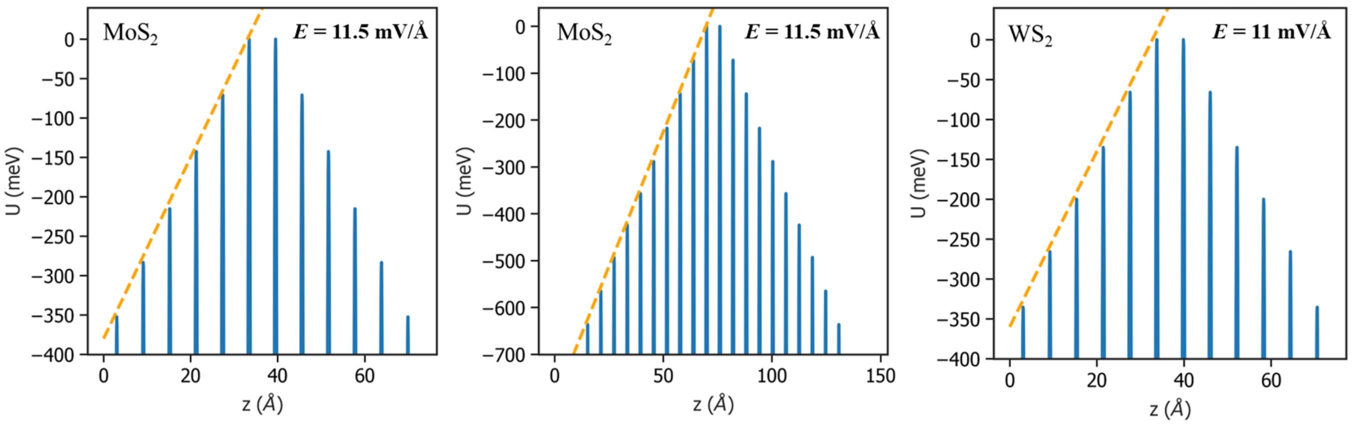

Supplementary Figure 1. **DFT-calculated potential drop and intrinsic electric field for MoS_2 mTB structures.** Calculated for 6 layers (left) and 12 layers (middle), as well as WS_2 mTB structures with 6 layers (right) in adjacent domains. The value of intrinsic electric field (mV/Å) is consistent between different supercell sizes.

[1] K. D. Bronsema, J. L. De Boer, and F. Jellinek, On the structure of molybdenum diselenide and disulfide, Zeitschrift für anorganische und allgemeine Chemie **540**, 15–17 (1986).

* vladimir.falko@manchester.ac.uk

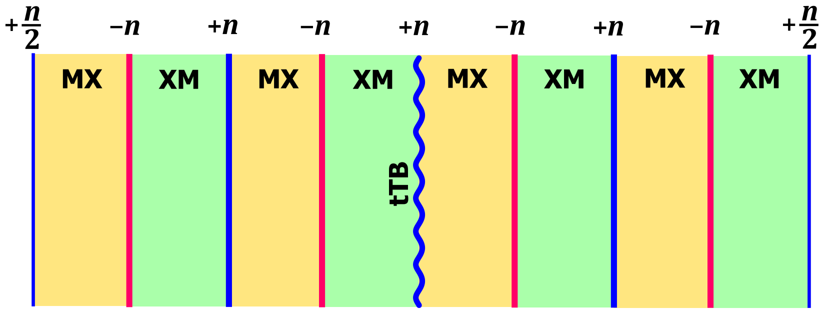

Supplementary Figure 2. **Schematic depiction of charge accumulation at internal TBs and surfaces.** Alternating FE polarisation domains hold alternating charge densities at aligned (mTBs) and twisted (tTB) twin boundaries; surfaces hold half this value.

Supplementary Table II. **Accumulation layer energies and Wigner paramters.** (Left) Ground state energy, quantum well depth, binding energy and Fermi energy for holes at mTBs and tTBs. (Right) Wigner parameter for holes at various twin boundaries.

h	ϵ_0 [meV]	U_∞ [meV]	ϵ_b [meV]	ϵ_F [meV]	r_s mTB & tTB	r_s Surface
MoS_2	60.4	88.7	28.4	27.7	2.6	5.8
WS_2	61.8	90.9	29.1	27.9	2.7	5.7
$MoSe_2$	45.7	67.1	21.5	21.5	2.8	7.0
WSe_2	47.9	70.5	22.5	23.4	2.7	6.5
$MoTe_2$	35.2	51.8	16.6	19.6	2.6	8.0

[2] W. Schutte, J. De Boer, and F. Jellinek, Crystal structures of tungsten disulfide and diselenide, Journal of Solid State Chemistry **70**, 207–209 (1987).

[3] D. Puotinen and R. Newnham, The crystal structure of mote2, Acta Crystallographica **14**, 691 (1961).

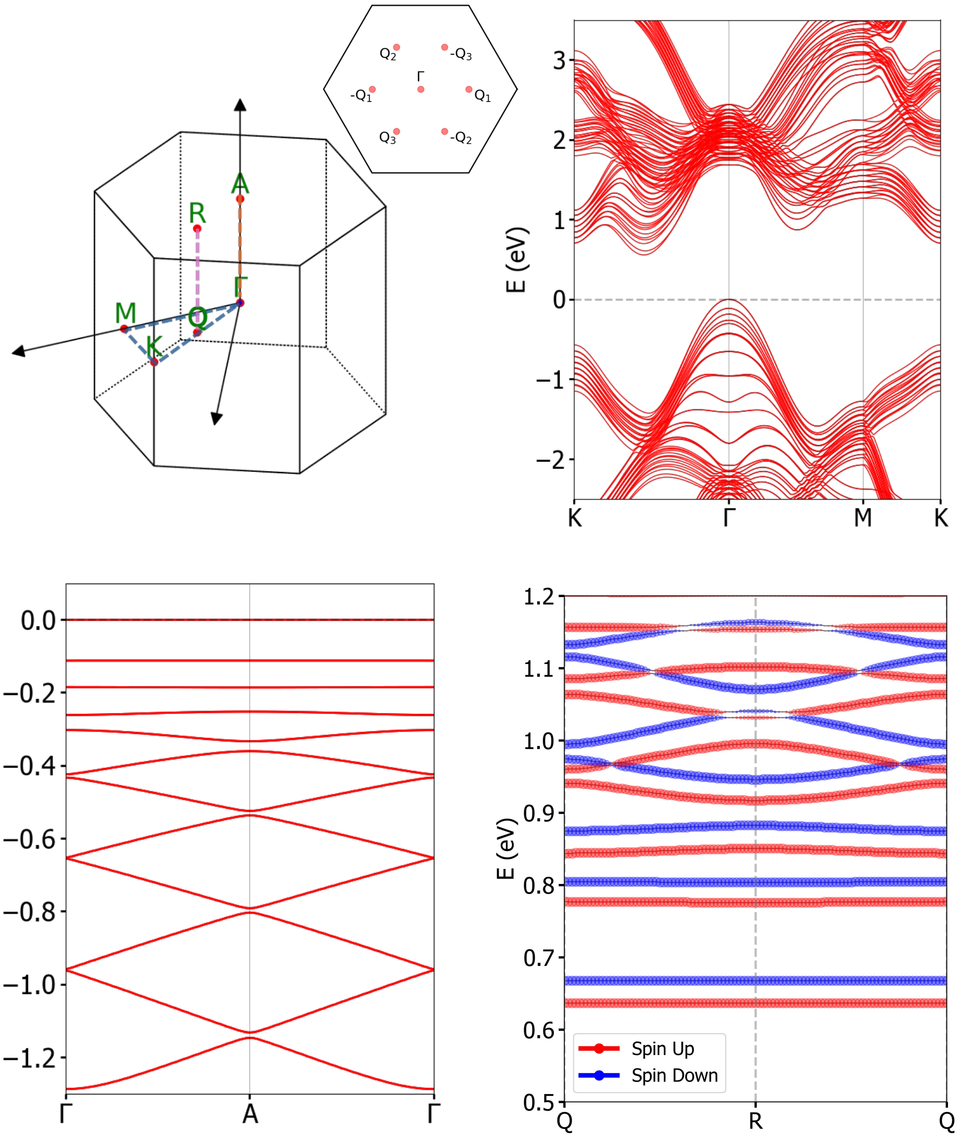

Supplementary Figure 3. **DFT-calculated electronic band structure of a 3R-MoS_2 crystal containing adjacent, periodic 6 layer MX and 6 layer XM twins.** Top left: paths in k-space and labelling of high-symmetry points adopted in this work. The c-axis direction has been shortened for visual clarity. Inset: top-down view of TMD Brillouin zone, illustrating 6 degenerate Q-points which occur as Kramers' pairs. Top right: band structure projected onto the $k_z = 0$ plane. Bottom: along the k_z-axis drawn through Γ-point (middle) and Q-point (right). For Q-R dispersion, spin orientation in spin-split bands is identified by colour, with opposite spin-splitting occurring at the $\pm Q_i$ points. This band structure data was used to determine the in-plane and c-axis masses for Γ-point holes and Q-point electrons.

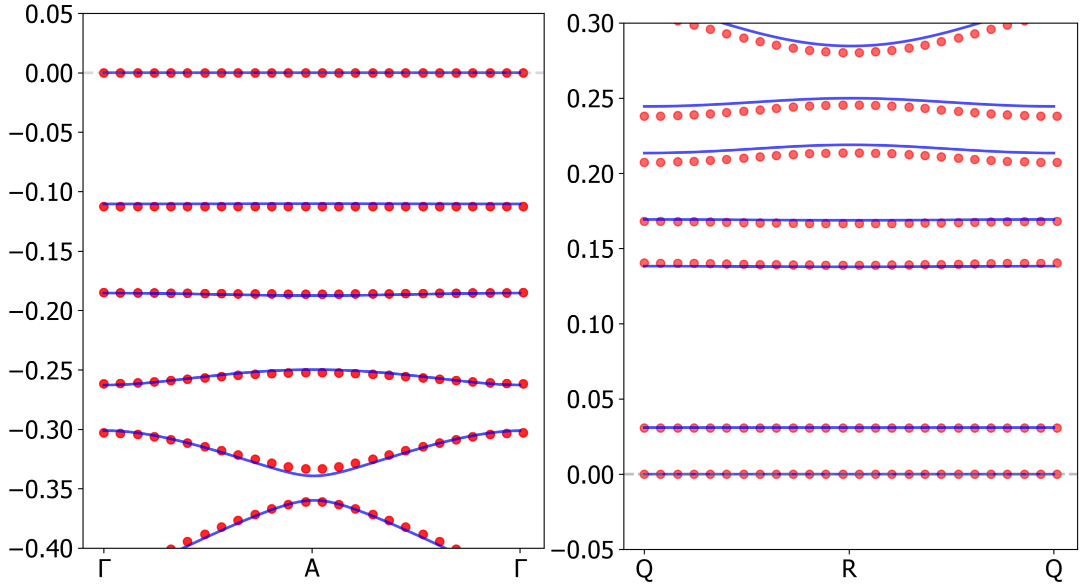

Supplementary Figure 4. **Out-of-plane mass fittings for 3R-TMDs.** DFT k_z dispersion of 3R-MoS_2 6-MX/6-XM periodic twin structure near the valence (left) and conduction (right) band edges [dots] in comparison with dispersion of an electron with effective masses shown in Supplementary Information Table I subjected to in the equivalent sawtooth potential [lines].

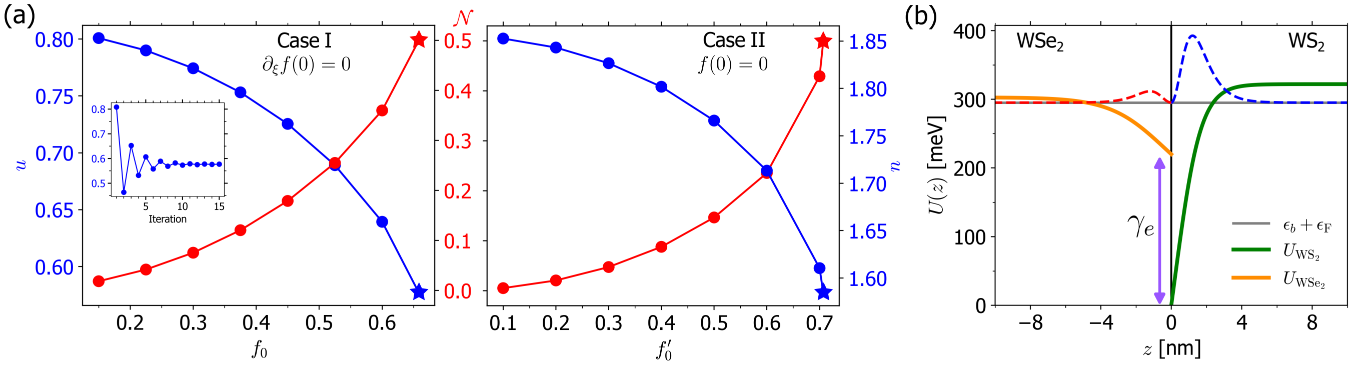

Supplementary Figure 5. **Shooting procedure and hetero-twin charge redistribution.** (a) Demonstration of the two-parameter shooting procedure used to solve Eq. 2, described in Methods. For each value of f_0 in case I (f_0' in case II), solution was found for $f(\xi)$ and u (blue dots), and a normalisation factor $\mathcal{N}$ was evaluated (red dots). This procedure started from f_0 and f_0' producing a small value of $\mathcal{N}$, and it was repeated until $\mathcal{N}$ reached the targeted $\frac{1}{2}$ value, marked by a star. For comparison, inset illustrates the convergence of solution obtained by using an alternative iterative approach. (b) Potential profile for n-type WSe_2/WS_2 twin boundary calculated from the universal solutions to the Thomas-Fermi problem and charge redistribution due to band edge mismatch γ_e. The electron density profiles are indicated by dashed lines, which are determined by $\tilde{n}_1|\psi_1|^2$ and $\tilde{n}_2|\psi_2|^2$ on WSe_2 and WS_2, respectively.
