# Supplementary material for: Two-dimensional electrons at mirror and twistronic twin boundaries in van der Waals ferroelectrics
Source: Nat Commun. 2024 Aug 9;15:6838. doi: 10.1038/s41467-024-51176-1 (PMC11316064; doi:10.1038/s41467-024-51176-1)
Supplement: Supplementary file 3 — Description of Additional Supplementary Files [file 41467_2024_51176_MOESM3_ESM.pdf]

Description of Additional Supplementary Files

Supplementary Data 1. Optimised DFT structures of mirror twin boundaries in 3R-TMDs.
