# Supplementary material for: Two-dimensional electrons at mirror and twistronic twin boundaries in van der Waals ferroelectrics
Source: Nat Commun. 2024 Aug 9;15:6838. doi: 10.1038/s41467-024-51176-1 (PMC11316064; doi:10.1038/s41467-024-51176-1)
Supplement: Supplementary file 4 — Supplementary Data 1 [file 41467_2024_51176_MOESM4_ESM.pdf]

Supplementary Data: Two-dimensional electrons at mirror and twistronic twin boundaries in van der Waals ferroelectrics

James G. McHugh,[1,2] Xue Li,[1,2] Isaac Soltero,[1,2] and Vladimir I. Fal'ko[1,2,3,*]

[1]*Department of Physics and Astronomy, University of Manchester. Oxford Road, Manchester, M13 9PL, United Kingdom*
[2]*National Graphene Institute, University of Manchester. Booth St. E., Manchester, M13 9PL, United Kingdom*
[3]*Henry Royce Institute for Advanced Materials, University of Manchester, Oxford Road, Manchester, M13 9PL, United Kingdom*

DFT-OPTIMISED STRUCTURES

Structures of 6, 9 and 12-layer MoS_2, 6-layer WS_2, $MoSe_2$, WSe_2 and 9-layer $MoTe_2$ mTB supercells are included below.


```
    36
Lattice="   3.153952144  0.000000000  0.000000000
-1.576976072   2.731402679   0.000000000
0.000000000  0.000000000  72.943186090"
Mo            0.0000000000       -0.0000000000       -0.0000000000
S             1.5769760732        0.9104675549        1.5664106138
S             1.5769760732        0.9104675549       -1.5664106138
Mo            0.0000000000        1.8209351197        6.0781920921
S             1.5769760732        2.7314026746        7.6452753087
S             1.5769760732        2.7314026746        4.5127620776
Mo            0.0000000000        3.6418702393       12.1567200650
S             1.5769760732        4.5523377942       13.7237363703
S             1.5769760732        4.5523377942       10.5912607588
Mo            0.0000000000        0.0000000000       18.2353039544
S             1.5769760732        0.9104675549       19.8022895803
S             1.5769760732        0.9104675549       16.6698452525
Mo            0.0000000000        1.8209351197       24.3138933532
S             1.5769760732        2.7314026746       25.8808746001
S             1.5769760732        2.7314026746       22.7484728441
Mo            0.0000000000        3.6418702393       30.3924477486
S             1.5769760732        4.5523377942       31.9594493756
S             1.5769760732        4.5523377942       28.8271116258
Mo            0.0000000000       -0.0000000000       36.4715930452
S             1.5769760732        0.9104675549       38.0375933947
S             1.5769760732        0.9104675549       34.9055926957
Mo           -0.0000000000       -1.8209351197       42.5507383418
S             1.5769760732       -0.9104675549       44.1160744646
S             1.5769760732       -0.9104675549       40.9837367148
Mo           -0.0000000000       -3.6418702393       48.6292927372
S             1.5769760732       -2.7314026746       50.1947132463
S             1.5769760732       -2.7314026746       47.0623114903
Mo           -0.0000000000        0.0000000000       54.7078821360
S             1.5769760732        0.9104675549       56.2733408379
S             1.5769760732        0.9104675549       53.1408965101
Mo           -0.0000000000       -1.8209351197       60.7864660254
S             1.5769760732       -0.9104675549       62.3519253316
```


* vladimir.falko@manchester.ac.uk

S	1.5769760732	-0.9104675549	59.2194497201
Mo	-0.0000000000	-3.6418702393	66.8649939983
S	1.5769760732	-2.7314026746	68.4304240128
S	1.5769760732	-2.7314026746	65.2979107817

```
    54
Lattice="3.153742075  0.000000000  0.000000000
-1.576871038   2.731220754   0.000000000
0.000000000  0.000000000 109.479636688"
Mo            0.0000000000       -0.0000000000       -0.0000000000
S             1.5768710387        0.9104069132        1.5664265079
S             1.5768710387        0.9104069132       -1.5664265079
Mo            0.0000000000        1.8208138363        6.0816351155
S             1.5768710387        2.7312207495        7.6489131539
S             1.5768710387        2.7312207495        4.5162976877
Mo            0.0000000000        3.6416276726       12.1637576269
S             1.5768710387        4.5520345858       13.7310470422
S             1.5768710387        4.5520345858       10.5984170673
Mo            0.0000000000       -0.0000000000       18.2459529700
S             1.5768710387        0.9104069132       19.8132447395
S             1.5768710387        0.9104069132       16.6806105755
Mo            0.0000000000        1.8208138363       24.3281495157
S             1.5768710387        2.7312207495       25.8954405059
S             1.5768710387        2.7312207495       22.7628087593
Mo            0.0000000000        3.6416276726       30.4103695765
S             1.5768710387        4.5520345858       31.9776598593
S             1.5768710387        4.5520345858       28.8450279927
Mo            0.0000000000        0.0000000000       36.4925586780
S             1.5768710387        0.9104069132       38.0598499505
S             1.5768710387        0.9104069132       34.9272169820
Mo            0.0000000000        1.8208138363       42.5747556025
S             1.5768710387        2.7312207495       44.1420470558
S             1.5768710387        2.7312207495       41.0094139360
Mo            0.0000000000        3.6416276726       48.6569557045
S             1.5768710387        4.5520345858       50.2242424411
S             1.5768710387        4.5520345858       47.0916146730
Mo           -0.0000000000       -0.0000000000       54.7398183439
S             1.5768710387        0.9104069132       56.3060023404
S             1.5768710387        0.9104069132       53.1736343474
Mo           -0.0000000000       -1.8208138363       60.8226809833
S             1.5768710387       -0.9104069132       62.3880220148
S             1.5768710387       -0.9104069132       59.2553942466
Mo           -0.0000000000       -3.6416276726       66.9048810853
S             1.5768710387       -2.7312207495       68.4702227518
S             1.5768710387       -2.7312207495       65.3375896320
Mo            0.0000000000        0.0000000000       72.9870780098
S             1.5768710387        0.9104069132       74.5524197058
S             1.5768710387        0.9104069132       71.4197867373
Mo           -0.0000000000       -1.8208138363       79.0692671113
S             1.5768710387       -0.9104069132       80.6346086951
S             1.5768710387       -0.9104069132       77.5019768285
Mo           -0.0000000000       -3.6416276726       85.1514871720
S             1.5768710387       -2.7312207495       86.7168279285
S             1.5768710387       -2.7312207495       83.5841961819
Mo           -0.0000000000        0.0000000000       91.2336837178
S             1.5768710387        0.9104069132       92.7990261123
S             1.5768710387        0.9104069132       89.6663919483
Mo           -0.0000000000       -1.8208138363       97.3158790609
S             1.5768710387       -0.9104069132       98.8812196204
S             1.5768710387       -0.9104069132       95.7485896456
Mo           -0.0000000000       -3.6416276726      103.3980015723
S             1.5768710387       -2.7312207495      104.9633390000
S             1.5768710387       -2.7312207495      101.8307235339
```

```
    72
Lattice="3.195660494   0.000000000   0.000000000
-1.597830247   2.767523170  0.000000000
0.000000000  0.000000000 149.453149031"
Mo            0.0000000000        0.0000000000        0.0000000000
S             1.5978302481        0.9225077183        1.5682873375
S             1.5978302481        0.9225077183       -1.5682873375
Mo           -0.0000000000        1.8450154466        6.2267860733
S             1.5978302481        2.7675231649        7.7956345532
S             1.5978302481        2.7675231649        4.6592576296
Mo            0.0000000000        3.6900308932       12.4539902206
S             1.5978302481        4.6125386115       14.0228435562
S             1.5978302481        4.6125386115       10.8864579782
Mo           -0.0000000000       -0.0000000000       18.6812025844
S             1.5978302481        0.9225077183       20.2500557589
S             1.5978302481        0.9225077183       17.1136698738
Mo           -0.0000000000        1.8450154466       24.9084166075
S             1.5978302481        2.7675231649       26.4772714849
S             1.5978302481        2.7675231649       23.3408864010
Mo            0.0000000000        3.6900308932       31.1356329192
S             1.5978302481        4.6125386115       32.7044867285
S             1.5978302481        4.6125386115       29.5681023377
Mo           -0.0000000000        0.0000000000       37.3628475878
S             1.5978302481        0.9225077183       38.9317016658
S             1.5978302481        0.9225077183       35.7953154177
Mo           -0.0000000000        1.8450154466       43.5900620510
S             1.5978302481        2.7675231649       45.1589157448
S             1.5978302481        2.7675231649       42.0225307349
Mo            0.0000000000        3.6900308932       49.8172750849
S             1.5978302481        4.6125386115       51.3861288073
S             1.5978302481        4.6125386115       48.2497438429
Mo           -0.0000000000       -0.0000000000       56.0444893239
S             1.5978302481        0.9225077183       57.6133432979
S             1.5978302481        0.9225077183       54.4769595424
Mo           -0.0000000000        1.8450154466       62.2717039740
S             1.5978302481        2.7675231649       63.8405577516
S             1.5978302481        2.7675231649       60.7041744856
Mo            0.0000000000        3.6900308932       68.4989155552
S             1.5978302481        4.6125386115       70.0677643410
S             1.5978302481        4.6125386115       66.9313863077
Mo            0.0000000000       -0.0000000000       74.7265745154
S             1.5978302481        0.9225077183       76.2946552794
S             1.5978302481        0.9225077183       73.1584937514
Mo            0.0000000000       -1.8450154466       80.9542334756
S             1.5978302481       -0.9225077183       82.5217627232
S             1.5978302481       -0.9225077183       79.3853846899
Mo           -0.0000000000       -3.6900308932       87.1814450569
S             1.5978302481       -2.7675231649       88.7489745453
S             1.5978302481       -2.7675231649       85.6125912793
Mo           -0.0000000000       -0.0000000000       93.4086597070
S             1.5978302481        0.9225077183       94.9761894885
S             1.5978302481        0.9225077183       91.8398057329
Mo            0.0000000000       -1.8450154466       99.6358739460
S             1.5978302481       -0.9225077183      101.2034051580
S             1.5978302481       -0.9225077183       98.0670202236
Mo           -0.0000000000       -3.6900308932      105.8630869799
S             1.5978302481       -2.7675231649      107.4306182660
S             1.5978302481       -2.7675231649      104.2942333161
```

Mo	-0.0000000000	-0.0000000000	112.0903014431
S	1.5978302481	0.9225077183	113.6578335832
S	1.5978302481	0.9225077183	110.5214473950
Mo	0.0000000000	-1.8450154466	118.3175161117
S	1.5978302481	-0.9225077183	119.8850466632
S	1.5978302481	-0.9225077183	116.7486623323
Mo	-0.0000000000	-3.6900308932	124.5447324234
S	1.5978302481	-2.7675231649	126.1122625999
S	1.5978302481	-2.7675231649	122.9758775760
Mo	-0.0000000000	0.0000000000	130.7719464465
S	1.5978302481	0.9225077183	132.3394791271
S	1.5978302481	0.9225077183	129.2030933019
Mo	0.0000000000	-1.8450154466	136.9991588103
S	1.5978302481	-0.9225077183	138.5666910227
S	1.5978302481	-0.9225077183	135.4303055046
Mo	-0.0000000000	-3.6900308932	143.2263629576
S	1.5978302481	-2.7675231649	144.7938913714
S	1.5978302481	-2.7675231649	141.6575145077

```
    36
Lattice="3.158217917  0.000000000  0.000000000
-1.579108959   2.735096947   0.000000000
0.000000000  0.000000000  73.595706242"
W             0.0000000000        0.0000000000       -0.0000000000
S             1.5791089597        0.9116989775        1.5759859806
S             1.5791089597        0.9116989775       -1.5759859806
W             0.0000000000        1.8233979649        6.1325721320
S             1.5791089597        2.7350969424        7.7090706489
S             1.5791089597        2.7350969424        4.5575506622
W            -0.0000000000        3.6467959298       12.2654726961
S             1.5791089597        4.5584949073       13.8419806419
S             1.5791089597        4.5584949073       10.6904459856
W             0.0000000000        0.0000000000       18.3984339530
S             1.5791089597        0.9116989775       19.9749440562
S             1.5791089597        0.9116989775       16.8234057955
W             0.0000000000        1.8233979649       24.5314012062
S             1.5791089597        2.7350969424       26.1079108268
S             1.5791089597        2.7350969424       22.9563745116
W            -0.0000000000        3.6467959298       30.6643974157
S             1.5791089597        4.5584949073       32.2409052927
S             1.5791089597        4.5584949073       29.0893687266
W            -0.0000000000       -0.0000000000       36.7978531208
S             1.5791089597        0.9116989775       38.3733864230
S             1.5791089597        0.9116989775       35.2223198187
W            -0.0000000000       -1.8233979649       42.9313088260
S             1.5791089597       -0.9116989775       44.5063375150
S             1.5791089597       -0.9116989775       41.3548009490
W             0.0000000000       -3.6467959298       49.0643050355
S             1.5791089597       -2.7350969424       50.6393317301
S             1.5791089597       -2.7350969424       47.4877954149
W            -0.0000000000        0.0000000000       55.1972722887
S             1.5791089597        0.9116989775       56.7723004462
S             1.5791089597        0.9116989775       53.6207621855
W            -0.0000000000       -1.8233979649       61.3302335456
S             1.5791089597       -0.9116989775       62.9052602560
S             1.5791089597       -0.9116989775       59.7537255998
W             0.0000000000       -3.6467959298       67.4631341096
S             1.5791089597       -2.7350969424       69.0381555795
S             1.5791089597       -2.7350969424       65.8866355928
```

```
36
Lattice="3.285637653  0.000000000   0.000000000
-1.642818827   2.845445675  0.000000000
0.000000000  0.000000000  77.343985377"
Mo          -0.0000000000       -0.0000000000       -0.0000000000
Se           1.6428188275        0.9484818865        1.6713656831
Se           1.6428188275        0.9484818865       -1.6713656831
Mo           0.0000000000        1.8969637834        6.4446721894
Se           1.6428188275        2.8454456700        8.1172012643
Se           1.6428188275        2.8454456700        4.7748459525
Mo          -0.0000000000        3.7939275669       12.8898298081
Se           1.6428188275        4.7424094534       14.5623656849
Se           1.6428188275        4.7424094534       11.2200049420
Mo           0.0000000000        0.0000000000       19.3350947163
Se           1.6428188275        0.9484818865       21.0076358666
Se           1.6428188275        0.9484818865       17.6652641534
Mo           0.0000000000        1.8969637834       25.7804011214
Se           1.6428188275        2.8454456700       27.4529390295
Se           1.6428188275        2.8454456700       24.1105736275
Mo          -0.0000000000        3.7939275669       32.2257487350
Se           1.6428188275        4.7424094534       33.8982852888
Se           1.6428188275        4.7424094534       30.5559204742
Mo          -0.0000000000       -0.0000000000       38.6719926884
Se           1.6428188275        0.9484818865       40.3429686702
Se           1.6428188275        0.9484818865       37.0010167066
Mo          -0.0000000000       -1.8969637834       45.1182366417
Se           1.6428188275       -0.9484818865       46.7880649027
Se           1.6428188275       -0.9484818865       43.4457000880
Mo           0.0000000000       -3.7939275669       51.5635842555
Se           1.6428188275       -2.8454456700       53.2334117494
Se           1.6428188275       -2.8454456700       49.8910463474
Mo          -0.0000000000       -0.0000000000       58.0088906606
Se           1.6428188275        0.9484818865       59.6787212235
Se           1.6428188275        0.9484818865       56.3363495103
Mo          -0.0000000000       -1.8969637834       64.4541555688
Se           1.6428188275       -0.9484818865       66.1239804348
Se           1.6428188275       -0.9484818865       62.7816196920
Mo           0.0000000000       -3.7939275669       70.8993131875
Se           1.6428188275       -2.8454456700       72.5691394244
Se           1.6428188275       -2.8454456700       69.2267841126
```

```
    36
Lattice="3.158217917  0.000000000  0.000000000
-1.579108959   2.735096947   0.000000000
0.000000000  0.000000000  73.595706242"
W            -0.0000000000       -0.0000000000        0.0000000000
Se            1.6432943663        0.9487564391        1.6820505511
Se            1.6432943663        0.9487564391       -1.6820505511
W             0.0000000000        1.8975128884        6.4794586243
Se            1.6432943663        2.8462693275        8.1623183190
Se            1.6432943663        2.8462693275        4.7989194758
W             0.0000000000        3.7950257768       12.9596109680
Se            1.6432943663        4.7437822159       14.6424851726
Se            1.6432943663        4.7437822159       11.2790600209
W            -0.0000000000       -0.0000000000       19.4398067683
Se            1.6432943663        0.9487564391       21.1226725867
Se            1.6432943663        0.9487564391       17.7592659237
W             0.0000000000        1.8975128884       25.9200039234
Se            1.6432943663        2.8462693275       27.6028798619
Se            1.6432943663        2.8462693275       24.2394549415
W             0.0000000000        3.7950257768       32.4001921078
Se            1.6432943663        4.7437822159       34.0830648185
Se            1.6432943663        4.7437822159       30.7196412334
W            -0.0000000000       -0.0000000000       38.8811643818
Se            1.6432943663        0.9487564391       40.5624932852
Se            1.6432943663        0.9487564391       37.1998354785
W             0.0000000000       -1.8975128884       45.3621366558
Se            1.6432943663       -0.9487564391       47.0426875303
Se            1.6432943663       -0.9487564391       43.6792639451
W            -0.0000000000       -3.7950257768       51.8423248403
Se            1.6432943663       -2.8462693275       53.5228738221
Se            1.6432943663       -2.8462693275       50.1594489018
W            -0.0000000000       -0.0000000000       58.3225219953
Se            1.6432943663        0.9487564391       60.0030628400
Se            1.6432943663        0.9487564391       56.6396561770
W             0.0000000000       -1.8975128884       64.8027177957
Se            1.6432943663       -0.9487564391       66.4832687428
Se            1.6432943663       -0.9487564391       63.1198435910
W            -0.0000000000       -3.7950257768       71.2828701394
Se            1.6432943663       -2.8462693275       72.9634092878
Se            1.6432943663       -2.8462693275       69.6000104447
```

54
Lattice="3.5899501707121013 0.000000000 0.000000000
-1.794975085356221 3.108988046156962 0.000000000
0.000000000 0.000000000 128.28597470532145"
Mo 0.0000000000 0.0000000000 0.0000000000
Te 1.7949750800 1.0363293500 1.8125813800
Te 1.7949750800 1.0363293500 -1.8125813800
Mo 0.0000000000 2.0726587000 7.1281649300
Te 1.7949750800 3.1089880500 8.9424301400
Te 1.7949750800 3.1089880500 5.3179265300
Mo 0.0000000000 4.1453173900 14.2552191300
Te 1.7949750800 5.1816467500 16.0691922500
Te 1.7949750800 5.1816467500 12.4447154700
Mo 0.0000000000 0.0000000000 21.3793309300
Te 1.7949750800 1.0363293500 23.1935565300
Te 1.7949750800 1.0363293500 19.5689674900
Mo 0.0000000000 2.0726587000 28.5053583200
Te 1.7949750800 3.1089880500 30.3195936500
Te 1.7949750800 3.1089880500 26.6951599100
Mo 0.0000000000 4.1453173900 35.6332248100
Te 1.7949750800 5.1816467500 37.4472194300
Te 1.7949750800 5.1816467500 33.8229358600
Mo 0.0000000000 0.0000000000 42.7597506500
Te 1.7949750800 1.0363293500 44.5737271600
Te 1.7949750800 1.0363293500 40.9494548000
Mo 0.0000000000 2.0726587000 49.8849464500
Te 1.7949750800 3.1089880500 51.6988891000
Te 1.7949750800 3.1089880500 48.0744922800
Mo 0.0000000000 4.1453173900 57.0122809900
Te 1.7949750800 5.1816467500 58.8264539400
Te 1.7949750800 5.1816467500 55.2019043300
Mo 0.0000000000 0.0000000000 64.1429873500
Te 1.7949750800 1.0363293500 65.9550884900
Te 1.7949750800 1.0363293500 62.3308862200
Mo 0.0000000000 -2.0726587000 71.2736937100
Te 1.7949750800 -1.0363293500 73.0840703800
Te 1.7949750800 -1.0363293500 69.4595207600
Mo 0.0000000000 -4.1453173900 78.4010282600
Te 1.7949750800 -3.1089880500 80.2114824200
Te 1.7949750800 -3.1089880500 76.5870856100
Mo 0.0000000000 0.0000000000 85.5262240500
Te 1.7949750800 1.0363293500 87.3365199000
Te 1.7949750800 1.0363293500 83.7122475400
Mo 0.0000000000 -2.0726587000 92.6527499000
Te 1.7949750800 -1.0363293500 94.4630388400
Te 1.7949750800 -1.0363293500 90.8387552800
Mo 0.0000000000 -4.1453173900 99.7806163800
Te 1.7949750800 -3.1089880500 101.5908148000
Te 1.7949750800 -3.1089880500 97.9663810600
Mo 0.0000000000 0.0000000000 106.9066437700
Te 1.7949750800 1.0363293500 108.7170072100
Te 1.7949750800 1.0363293500 105.0924181700
Mo 0.0000000000 -2.0726587000 114.0307555700
Te 1.7949750800 -1.0363293500 115.8412592400
Te 1.7949750800 -1.0363293500 112.2167824600
Mo 0.0000000000 -4.1453173900 121.1578097800
Te 1.7949750800 -3.1089880500 122.9680481800
Te 1.7949750800 -3.1089880500 119.3435445700
